# Eigenvector Spatial Filtering Regression Modeling of Ground PM_2.5_ Concentrations Using Remotely Sensed Data

**DOI:** 10.3390/ijerph15061228

**Published:** 2018-06-11

**Authors:** Jingyi Zhang, Bin Li, Yumin Chen, Meijie Chen, Tao Fang, Yongfeng Liu

**Affiliations:** 1 School of Resource and Environment Science, Wuhan University, Wuhan 430079, China; jyzhang0@whu.edu.cn (J.Z.); chen_meijie@whu.edu.cn (M.C.); fountaintop@whu.edu.cn (T.F.); 2 Department of Geography and Environmental Studies, Central Michigan University, Mount Pleasant, MI 48859, USA; li1b@cmich.edu; 3 Wuhan Geomatics Institute, Wuhan 430022, China; yfliu91@163.com

**Keywords:** fine particulate matter (PM_2.5_), spatial effect, eigenvector spatial filtering method, regression model

## Abstract

This paper proposes a regression model using the Eigenvector Spatial Filtering (ESF) method to estimate ground PM_2.5_ concentrations. Covariates are derived from remotely sensed data including aerosol optical depth, normal differential vegetation index, surface temperature, air pressure, relative humidity, height of planetary boundary layer and digital elevation model. In addition, cultural variables such as factory densities and road densities are also used in the model. With the Yangtze River Delta region as the study area, we constructed ESF-based Regression (ESFR) models at different time scales, using data for the period between December 2015 and November 2016. We found that the ESFR models effectively filtered spatial autocorrelation in the OLS residuals and resulted in increases in the goodness-of-fit metrics as well as reductions in residual standard errors and cross-validation errors, compared to the classic OLS models. The annual ESFR model explained 70% of the variability in PM_2.5_ concentrations, 16.7% more than the non-spatial OLS model. With the ESFR models, we performed detail analyses on the spatial and temporal distributions of PM_2.5_ concentrations in the study area. The model predictions are lower than ground observations but match the general trend. The experiment shows that ESFR provides a promising approach to PM_2.5_ analysis and prediction.

## 1. Introduction

PM_2.5_, particles with an aerodynamic diameter of 2.5 μm or less, are harmful to both the natural environment and human health. These small particles are responsible for such environmental issues as corrosion, soiling, damage to vegetation and especially, reduced visibility [1,2], as they are major air pollutants that cause haze [3,4]. Due to their small size, PM_2.5_ particles can penetrate deep into the lungs through breathing and seep into the blood system along with toxic substances on their surfaces, posing severe health risks to the human body. Exposure to PM_2.5_ for only a few days can bring about adverse health effects [5,6,7,8].

As the number of smog days surged in major cities across China in recent years, the Chinese government has become increasingly concerned about PM_2.5_ pollution and has added more ground stations to monitor this contaminant, from 496 stations in 2012 to 1436 in 2016. This is a substantial improvement but they are still far from enough. Ground monitoring stations remain sparse and distributed unevenly, making it difficult to perform detailed analyses on the spatial and temporal variation of PM_2.5_ concentrations in large areas [9]. To overcome the problem of data availability, remote sensing imagery with high spatial resolution is widely utilized in PM_2.5_ research.

Wang and Christopher compared Aerosol Optical Depth (AOD) data acquired by the Moderate Resolution Imaging Spectroradiometer (MODIS) sensor on the Terra and Aqua satellites with ground PM_2.5_ concentrations and found a good linear relationship between them [10]. Afterwards, other quantitative studies provided further evidence that AOD is correlated with PM_2.5_ concentration and linear regression models based on the PM_2.5_-AOD relationship were developed [11,12,13,14]. New AOD datasets with higher spatial resolution provide a better way for getting high-resolution data for measuring PM_2.5_ concentrations, which has led to the identification of more contributing covariates. For example, researchers found that surface temperature and pressure have an impact on particle generation and movement; therefore, they can influence PM_2.5_ concentrations [13,15,16,17]. NDVI, an indicator of vegetation coverage, is another influential factor as plants have the ability to absorb pollutants through their leaf surfaces and hence purify the air [18]. Planetary boundary layer height and relative humidity can influence the relationship between AOD and PM_2.5_ [19,20,21]. Besides, terrain and location of pollution sources such as roads and factories also influence PM_2.5_ distributions [22]. These covariates can all be applied to PM_2.5_ estimation models.

Multiple Linear Regression models are commonly applied to estimating PM_2.5_ concentrations because of their ease of use and strong applicability [23,24]. Generalized Linear Models and Generalized Additive Models have also been applied to the study of PM_2.5_ [25,26]. However, these models have the same shortcoming as they neglect spatial autocorrelation which exists in most geospatial processes and affects the performances of conventional regression models. Failure to take spatial effects into account would result in estimation bias and increase model uncertainty [17,27]. In addition, the relationships between PM_2.5_ concentration and its influencing factors are spatially heterogeneous, with the strength and nature of relationships varies with space [11]. Researchers have attempted to use Geographically Weighted Regression (GWR) to address this issue of spatial heterogeneity in PM_2.5_ modeling [20,28,29,30,31].

GWR is different from the global models in that it produces regression coefficients varying over a geographic landscape. The locational weighted approach fits the data well and sheds new light on the understanding of PM_2.5_, as GWR models can reveal where the strong and weak relationships lie. Land Use Regression (LUR) is another common modeling approach in PM_2.5_ estimation. It attempts to incorporate spatial effects by bringing geographical features such as land use, traffic and population into the model [32,33,34,35]. It does not however take spatial autocorrelation into account. Thus capturing spatial autocorrelation offers another venue for improvement.

Eigenvector spatial filtering (ESF) is proposed to account for spatial effects. It selects a subset of eigenvectors of a spatially weight matrix and adds them to the original regression model as new independent variables. The linear combination of these eigenvectors filters the spatial autocorrelation out of the observations, thus enabling model processes to proceed as if the observations were independent [36,37]. In this paper, we report an Eigenvector Spatial Filtering Regression (ESFR) model for estimating PM_2.5_ concentrations. Independent variables include Aerosol Optical Depth (AOD), Surface Temperature (ST), Relative Humidity (RH), Pressure (PS), Planetary Boundary Layer Height (PBLH), Normalized Difference Vegetation Index (NDVI), Digital Elevation Model (DEM), densities of roads (Road_Den_) and densities of factories (Fact_Den_), because these covariates are found to be closely associated with PM_2.5_ [22,38,39]. Except for the densities of roads and factories, data are all derived from satellite observations, which overcomes the geographic limitations and achieves a greater regional coverage [11]. Model results will be compared with those from Global Multiple Linear Regression (GMLR) models; model predictions will be mapped and analyzed to reveal the spatial and temporal characteristics of ground PM_2.5_ concentrations in China’s Yangtze River Delta Region.

## 2. Materials and Methods

### 2.1. Study Areas

**Figure 1:**
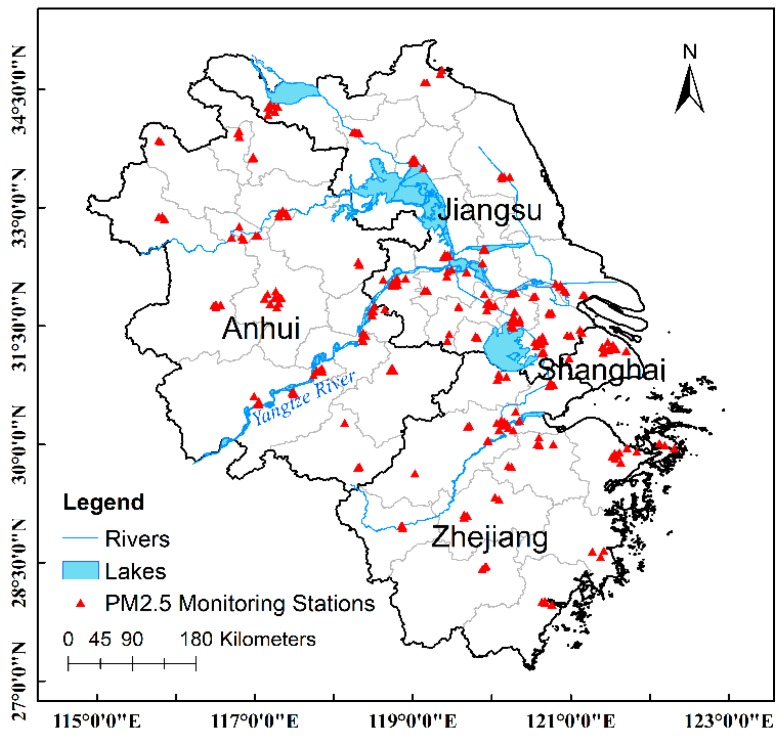
Study area and PM_2.5_ monitoring station locations.

The Yangtze River Delta (YRD) Region, composed of three provinces, Jiangsu, Anhui, and Zhejiang, as well as the Shanghai municipality, is used as a case study (Figure 1). The YRD region is located on China’s eastern coast, lying between around longitudes 115° and 123° E, and latitudes 27° and 35° N. This region has a land area of over 350,000 square kilometers and a population of around 220 million. It is China’s largest economic center. However, the rapid development was accompanied by environmental deterioration, particularly in air quality. In 2016, the annual PM_2.5_ concentration in this area was 47.4% greater than the national limit (35 μg/m^3^).

### 2.2. Ground PM_2.5_ Concentrations

The Qingyue Data Center (https://data.epmap.org/air/nations) provides air pollutants data collected from the China Environmental Monitoring Center (CEMC). Data are derived from ground monitoring sites, which include hourly and daily average mass concentrations of PM_2.5_ and other air pollutants. PM_2.5_ concentrations are measured by the tapered element oscillating microbalance (TEOM) method with an accuracy of ±0.5 μg/m^3^ for the daily average. PM_2.5_ measurements from 1 December 2015 to 30 November 2016 over the YRD Region (including 233 stations in total) were collected from this data center. Invalid data records, for example those with null or negative values, were removed from the dataset.

### 2.3. Remotely Sensed Data

MODIS provides daily 3 km resolution ambient AOD products, which will be used to generate PM_2.5_ raster grids. The MOD04_3k_v6 dataset was downloaded from the NASA website (https://search.earthdata.nasa.gov) and we extracted the Dark Target algorithm retrieved AOD in the study area. AOD data points with quality flag 0 were removed. The NDVI data were also MODIS products (MOD13A3) with a spatial resolution of 1 km. Satellite meteorological data were derived from the Goddard Earth Observing System Data Assimilation System (https://gmao.gsfc.nasa.gov/products/), which include Surface Temperature (ST), Relative Humidity (RH), Pressure (PS), and Planetary Boundary Layer Height (PBLH). Their spatial resolutions were 0.25° latitude × 0.3125° longitude. The Shuttle Radar Topography Mission (SRTM) DEM product was downloaded from the http://srtm.csi.cgiar.org website and its spatial resolution was 90 m.

### 2.4. Pollution Source Data

We extracted the roads networks from OpenStreetMap and obtained the factory locations from BaiduMap. They were used in the PM_2.5_ model specification as well as analysis of PM_2.5_ pollution causes. As PM_2.5_ pollution mainly comes from industrial emissions and motor vehicle exhaust [40,41], we simplified the roads networks by keeping only the types of roads used for motor vehicles, such as primary roads, secondary roads, trunks, raceways, motorways and railways. Duplicate factory points were removed, resulting in a total of 23,299 factory points in the study area.

### 2.5. Data Preprocessing

Several steps were followed to prepare the data for regression modeling. The first step is rescaling all data sets to the same spatial resolution of 3 kilometers, consistent with the AOD data. Because the meteorological data (ST, PS, RH and PBLH) have a coarser spatial resolution than the AOD, we interpolated them to 3 km using Ordinary Kriging with the spherical model. NDVI and DEM have a finer resolution than the AOD and they were therefore resampled into the 3 km grid using bilinear-interpolation. In the second step, we created the density grids for factories and roads at 3 km resolution as potential covariates. The point or line density in a grid cell is calculated as the number of points or the total length of lines falling in the buffer of 24 kilometers. To accommodate temporal analysis, annual, seasonal and monthly averages of each variable were calculated. In the final step, data values of the candidate independent variables, which are extracted from the raster grids, were assigned to the corresponding stations. Stations with null value were removed. In the end, we have 3301 records at annual, seasonal and monthly time scales in total, each record with a PM_2.5_ value and a vector of the covariates including AOD, ST, PS, PBLH, RH, NDVI, DEM, densities of factories and roads.

### 2.6. Spatial Regression with Eigenvector Spatial Filtering

Geographic variables often exhibit spatial autocorrelation, which affects the accuracy and uncertainty of the parameter estimates in regression models. Recent advances in spatial statistics provide several approaches to remedy the problem by including spatial autocorrelation in the classic statistical models [42,43]. Spatial autoregressive models, signified by the spatial lag and spatial error models in spatial econometrics, are now commonly used [44,45,46]. Meanwhile, Eigenvector Spatial Filtering Regression (ESFR), which represents spatial autocorrelation as a synthetic variable derived from a linear combination of selected eigenvectors of the spatial weights matrix, is gaining recognitions [47,48]. Equation (1) is the general form of the ESFR model [49]:

(1) Y=Xβ+Eα+ε

where ***Y*** is an n×1 vector of dependent variable, ***X*** is an n×p matrix containing independent variables, ***E*** is an n×k matrix containing *k* selected eigenvectors, ***α*** and ***β*** are the corresponding vectors of regression coefficients, and *ε* is a vector of i.i.d random errors.

The linear combination of the selected eigenvectors ***Eα*** filters the spatial autocorrelation out of the regression residuals. Because the eigenvectors are orthogonal and uncorrelated with each other, we are able to use them as synthetic variables in the regression model and estimate the parameters using conventional methods such as OLS, constructing models with improved accuracy and reduced uncertainty [37,50]. Researchers have begun to use ESF to model PM_2.5_ [51] where the independent variables are observations of other air pollutants at the same monitoring stations as PM_2.5_. The application of the model is limited as the number of monitoring stations is insufficient to cover a large area. In this paper, we use remotely sensed data with high resolution to build the ESFR model, making it more practical for PM_2.5_ analysis and prediction.

### 2.7. Model Specification, Assessment and Comparison

Estimating the ESFR model for PM_2.5_ concentration includes five steps: (1) construction of the spatial weights matrix for the study area; (2) calculation of the eigenvectors from the centered spatial weights matrix; (3) selections of eigenvectors using step-wise regression with all the covariates; (4) OLS estimation of the regression coefficients; (5) removal of the insignificant covariates in the obtained model and repeating steps (3) and (4) to construct the final ESFR model.

The spatial weights matrix **C_0_** for the stations can be topology-based as well as distance-based [47,52]. In this paper, **C_0_** is a distance-based matrix whose (*i*, *j*)-th element equals exp(–*d_i_*_,*j*_/*r*), where *d_i_*_,*j*_ is the Euclidean distance between stations *i* and *j*, and *r* is the longest distance in the minimum spanning tree covering stations. Subsequently, the spatial weights matrix C_0_ is centered as follows:

(2) $$C_{1}=\left(I-11^{T}/n\right)C_{0}\left(I-11^{T}/n\right)$$

where **1** is an n×1 vector of ones, which means **11**^T^ is an n×n matrix whose elements all equal one, and T denotes the matrix transpose operator, *n* is the number of monitoring stations and I is an n-dimension identity matrix. To proceed with the ESFR, eigenvectors are calculated for the centered matrix **C_1_**, followed by a selection process which often involves such variable selection methods as step-wise regression as well as the Least Absolute Shrinkage and Selection Operator (LASSO) [53,54,55]. Candidate eigenvectors were first calculated from the distance-based weights matrix for the study area (Figure 1). Since all of the variables have positive spatial autocorrelation, the subset of eigenvectors was initially formed through the criteria $(\lambda_{i}/\lambda_{1})\geq 0.10$ [48] which then was further selected along with the covariates through step-wise regression. The combination of eigenvector resulting in the highest R-squared is remained. The initial model is specified in the following form:

(3) $${\mathrm{PM}}_{2.5}=\beta_{0}+\beta_{1}AOD+\beta_{2}ST+\beta_{3}RH+\beta_{4}PBLH+\beta_{5}PS+\beta_{6}NDVI+\beta_{7}DEM+\beta_{8}Fact_{Den}+\beta_{9}Road_{Den}+E_{k}\beta_{k}+\varepsilon$$

where $\beta_{0}$ is the intercept, $\beta_{i}\;\left(i=1,\ldots ,9\right)$ are regression coefficients. $E_{k}$ is an n×k matrix of selected eigenvectors, $\beta_{k}$ is a k×1 vector of coefficients for the eigenvectors, ε is an n×1 error vector. The term $E_{k}\beta_{k}$ is the spatial filter, accounting for the spatial effects in PM_2.5_ distribution. After the eigenvectors are selected, OLS is used for estimating the model as specified in Equation (3).

Model performance will be assessed through the common metrics for OLS models, including Adjusted R^2^, Residual Standard Errors (RSE), Mean Absolute Percentage Error (MAPE) and Corrected Akaike Information Criterion (AICc). Residuals’ global Moran’s I was computed to validate if the spatial autocorrelation was filtered out of the residuals and the residuals were spatially random. To assess model fitting and prediction accuracy, leave-one-out cross validation (LOOCV) was conducted. We selects one station for validation and the remaining stations are used as the training data. This is repeated until every station is used as the validation data once. Then we calculate the Mean Squared Error (MSE) of the validation dataset to assess model estimation accuracy. For LOOCV of ESFR model, the R package “spmoran” provides functions for ESF-based spatial interpolation based on a minimization of expected error, which can be used to calculate the eigenvector for the stations to be predicted. The ESFR model is compared with the conventional non-spatial OLS model (denoted as Global Multiple Linear Regression, or GMLR), using the above performance metrics and cross-validations.

### 2.8. PM_2.5_ Distribution Mapping and Cause Analysis

The annual and seasonal ESFR models will be applied to generate the ground PM_2.5_ maps. As the covariates were all 3 km raster layers, we interpolated the selected eigenvectors into the 3 km grids and applied the ESFR model to producing PM_2.5_ maps. In this way, PM_2.5_ concentrations on the grid cells with no monitoring stations were computed and we obtained continuous annual and seasonal PM_2.5_ distribution maps in the YRD region. We can evaluate the air quality status as well as analyzing spatiotemporal characteristics of PM_2.5_ concentrations in the YRD region from a finer scale using these maps.

To explore the causes of the PM_2.5_ distribution, we introduced a measurement of pollution sources density, defined as the average of normalized point density of factory POIs and the line density of roads, as is shown in Equation (4):

(4) $$\mathrm{Pollution}\;\mathrm{Sources}\;\mathrm{Density}=\frac{1}{2}\left({\mathrm{Road}}_{\mathrm{Den}\_\mathrm{Norm}}+{\mathrm{Fact}}_{\mathrm{Den}\_\mathrm{Norm}}\right)$$

Road_Den_Norm_ is the normalized Road_Den_, Fact_Den_Norm_ is the normalized Fact_Den_. The normalization method for Road_Den_ and Fact_Den_ is shown in Equation (5):

(5) $$X_{Norm}=\left(X-Min\right)/\left(Max-Min\right)$$

*Max* and *Min* denote the maximum and minimum values of *X*. The pollution sources density map was compared to the PM_2.5_ maps for visual exploration of the relationship between them.

## 3. Results

### 3.1. Data Review and Pre-Analysis

#### 3.1.1. Dataset Summary

Table 1 is an overview of the original dataset. The average PM_2.5_ concentration in the YRD region is 51.6 μg/m^3^. The average AOD is 0.54. The average ST, PS, RH, PBLH and NDVI are 292.0 K, 1001.3 hpa, 69.7%, 389.7 m and 62.0%, respectively. The average elevation is 138.3 m. PM_2.5_ concentration in the study area is high on the whole according to the Chinese national standards (GB 3095—2012), with an annual average 47.4% greater than the national limit (35 μg/m^3^).

AOD: Aerosol Optical Depth; ST: Surface Temperature; PS: Pressure; RH: Relative Humidity; PBLH: Planetary Boundary Layer Height; NDVI: Normalized Difference Vegetation Index; Std.dev denotes Standard Deviation.

**Figure 2:**
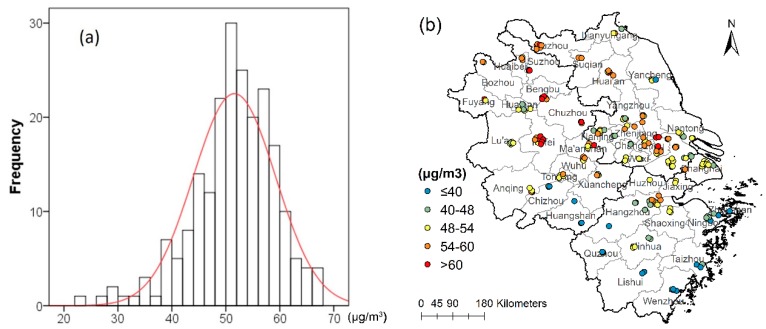
Histogram (**a**) and geographic distribution map (**b**) of the annual mean PM_2.5_ ground observations, the red curve is the standard normal curve.

Figure 2 shows the distribution characteristics of annual PM_2.5_ observations, which ranges from 23.2 to 67.9 μg/m^3^. The histogram (Figure 2a) indicates that the annual PM_2.5_ distribution is approximately normal. The PM_2.5_ observation map shows the spatial difference (Figure 2b): the high concentration clusters in Hefei and Bengbu of Anhui Province, while the low concentration is located in Huangshan and Zhoushan of Zhejiang Province.

**Figure 3:**
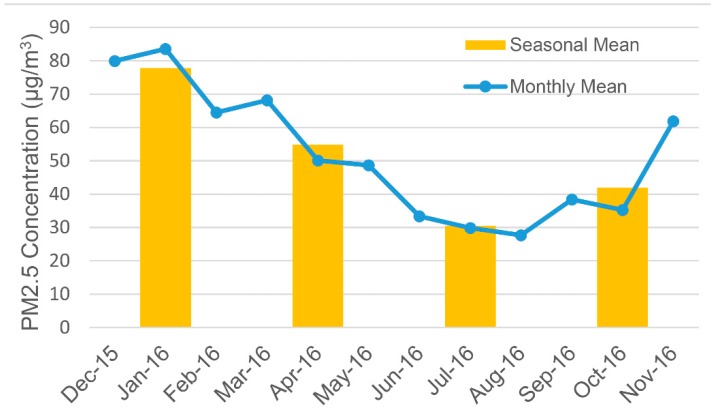
Seasonal and monthly mean PM_2.5_ concentrations calculated from ground observations.

Figure 3 depicts the monthly and seasonal mean PM_2.5_ concentrations calculated from ground observations. PM_2.5_ pollution is most serious in winter (77.9 μg/m^3^) followed by spring (54.9 μg/m^3^) and autumn (42.0 μg/m^3^). The summer average (30.5 μg/m^3^) is the lowest. The monthly averages from April to October are below the annual average while the other months are above the mean. It can be seen that the monthly average line is U-shaped and the bottom is reached in August, which denotes the best air quality in the year.

#### 3.1.2. Correlation Analysis

Table 2 shows the Pearson Correlation Coefficients (PCC) between the annual and seasonal PM_2.5_ concentration averages and the covariates. It can be seen from the annual measurements that PM_2.5_ concentration is moderately and positively correlated with AOD and PS while negatively correlated with PBLH, RH, DEM and ST. NDVI is weakly and negatively correlated with PM_2.5_. Fact_Den_ and Road_Den_ are weakly and positively correlated with PM_2.5_. There are variations in seasonal correlation results. Generally, AOD, PS, Fact_Den_ and Road_Den_ are positively correlated with PM_2.5_ while PBLH, RH, NDVI and DEM are negatively correlated with PM_2.5_. There are exceptions: some variables don’t have significant correlations with PM_2.5_ in certain period such as AOD in autumn, RH in summer, and Road_Den_ in autumn. The relationship between ST and PM_2.5_ is not stationary through time. They are positively correlated with PM_2.5_ in spring, negatively correlated in autumn and winter, and not correlated in summer.

Table 3 shows the monthly results are consistent with those of annual and seasonal analyses on the whole. However, opposite results appear in several months. For example, PBLH is not correlated with PM_2.5_ in May and June. NDVI does not have significant correlation with monthly PM_2.5_ values except in December, February, July and November. The relationship between ST and PM_2.5_ is even more complex, with the PCC varying from −0.591 to 0.235. PCC of RH varies from −0.550 to 0.228. Results show that the correlations between PM_2.5_ and covariates change with time, motivating us to construct PM_2.5_ models at multi time scales to avoid temporal effects. However, the PCC is limited in correlation analysis between PM_2.5_ and a certain variable because it can be influenced by other influential factors. Correlation analysis based on regression models can be more accurate.

#### 3.1.3. Spatial Autocorrelation Analysis

Since spatial effects have an impact on model accuracy and uncertainty, it is important to examine the nature and magnitude of spatial autocorrelation in the data. Table 4 shows the Moran’s I values of PM_2.5_ concentrations at different time scales, which are all positive, ranging from 0.296 to 0.610 and statistically significant, indicating the geographic distribution of PM_2.5_ is highly clustered. The magnitudes of spatial autocorrelation change at different time scales.

The Moran’s I of annual mean PM_2.5_ is 0.563. As for the seasonal time scale, the spatial autocorrelation of PM_2.5_ is strongest in autumn, followed by winter and spring, and becomes weakest in summer. At the monthly scale, spatial autocorrelation is strongest in March and November but weakest in May and June. Nevertheless, the prevailing presence of spatial autocorrelation across time scales suggests that model performances would be improved greatly if spatial information is incorporated in model specifications.

### 3.2. ESFR Model

ESFR models at annual, seasonal and monthly levels were estimated. Table 5 and Table 6 report the modeling results, including the coefficient estimates and the *p*-values of the ESFR models. For ease of comparison, the variables are standardized and standardized coefficients are given instead of the original coefficients. For insignificant variables removed in the initial modeling step, their coefficients and *p*-value are marked with ‘/’. The *p*-values indicate the significance of variables in PM_2.5_ modeling changes with time. In the annual ESFR model, the selected variables including AOD, ST, PS, RH, PBLH, NDVI, DEM and Fact_Den_ are all significant at α = 0.1 level. For the seasonal and monthly models, these variables have significance test results different from the annual model. AOD is not significant in Winter, Spring, Summer and some months such as December, January. RH is not significant in the Spring and January models. PBLH is not significant in May and November. Fact_Den_ is not significant in the December model. Although Road_Den_ is not selected in the annual model, it is significant in January and September.

Among the statistically significant variables, it can be seen from the standardized coefficients (denoted as Beta) that they have different effects on PM_2.5_ concentration. In the annual model, PM_2.5_ concentration is positively correlated with AOD, PS, RH and Fact_Den_ while negatively correlated with ST, PBLH, NDVI and DEM. For the seasonal and monthly models, the effects of PS, DEM, NDVI and Fact_Den_ on PM_2.5_ are consistent with the annual model. However, some variables present results opposite to the annual model. For example, the coefficient of AOD in October is −0.12, indicating a negative effect on PM_2.5_. The coefficients of RH in Summer, April, May and July indicate a negative effect on PM_2.5_. Besides, Road_Den_ has positive effect on PM_2.5_ according to the monthly models.

ESFR models have different performance in different periods. The annual ESFR model has the best fit with an adjusted R^2^ of 0.70, an LOOCV MSE of 19.2 and an AICc of 1255.8. ESFR model performs best in autumn (R^2^_adj_= 0.64) and worst in spring (R^2^_adj_ = 0.49). Among monthly models, ESFR model in November has the best performance (R^2^_adj_ = 0.73) and worst in June (R^2^_adj_ = 0.36). In December and January when PM_2.5_ pollution is often serious, the models also perform well.

Though all nine of the covariates are reported to be significant influential factors in previous PM_2.5_ studies, some of them are not significant in our case study. In most of the models, AOD, NDVI and Road_Den_ are not useful independent variables for PM_2.5_ estimation while PBLH and Fact_Den_ remained significant in most models. Because the relationships between these independent variables and PM_2.5_ change with space and time, our wide span of study area and time period may weaken their relationship and lead to insignificant coefficients.

### 3.3. Model Assessment and Comparison

#### 3.3.1. Model Fit

Table 7 shows the performance metrics of the annual and seasonal PM_2.5_ ESFR models. Performance indicators for the annual GMLR models are also given for comparison. The adjusted R^2^ of annual ESFR model reaches 0.70, 16.7% higher than the GMLR model. The RSE is 4.24 μg/m^3^ and the MAPE is 6.66%, which decreases by 12.7% and 13.5%, respectively, compared with the annual GMLR model. The AICc of ESFR model is lower than the GLMR model, which means there is less information loss in the ESFR model.

For the seasonal models, the autumn ESFR model has the best performance with an adjusted R^2^ 0.65, which increases by 34.1% over the GMLR model. Its RSE decreases 17.3%. Though the spring ESFR model is the worst among the four seasons, it increased 25.4% in adjusted R^2^ compared to the GMLR model. Its RSE decreases 8.5%. Considering that serious air pollution events often occur in winter and autumn, the ESFR model is of great significance for practical application.

**Figure 4:**
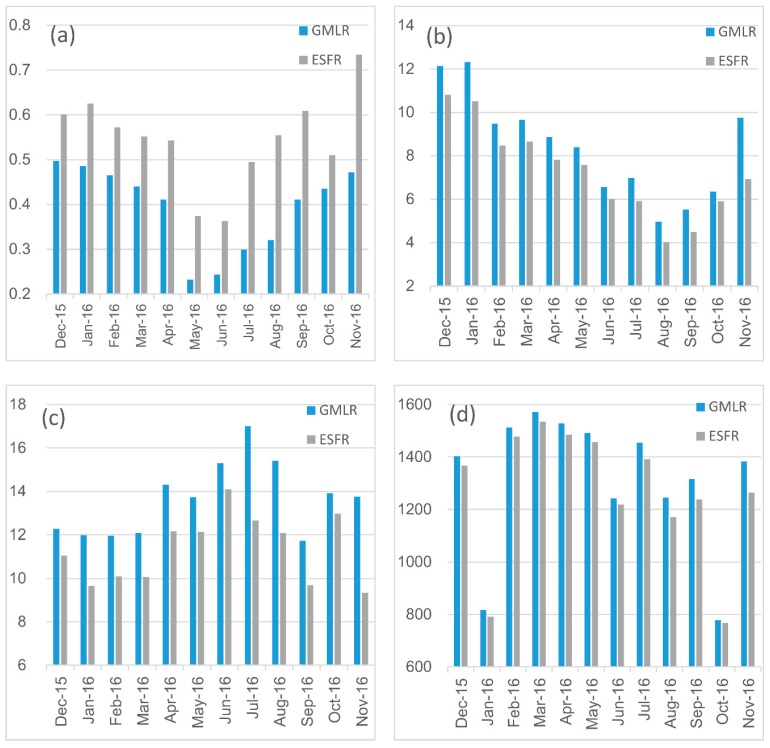
Adjusted R^2^ (**a**), RSE (**b**), MAPE (**c**) and AICc (**d**) comparisons among monthly PM_2.5_ GMLR and ESFR models.

The monthly results are shown in Figure 4. For the GMLR models, their adjusted R^2^ ranged from 0.23 to 0.50, with a mean value of 0.39. The RSE ranged from 4.97 to 12.32 μg/m^3^, with a mean value of 8.41 μg/m^3^. The MAPE ranged from 11.73% to 17.01%, with a mean value of 13.62%. The ESFR models have higher adjusted R^2^, lower RSE and MAPE than the GMLR models. The adjusted R^2^ ranged from 0.36 to 0.73, with a mean value of 0.54. The RSE ranged from 4.03 μg/m^3^ to 10.51 μg/m^3^, with a mean value of 7.26 μg/m^3^. The MAPE ranged from 9.34% to 14.10%, with a mean value of 11.33%. The AICc values of ESFR models are obviously lower than those of GMLR models. In conclusion, ESFR model has significant improvements on model fitting precision compared with GMLR model.

#### 3.3.2. Model Residuals Moran’s I

Table 8 shows the Moran’s I of model residuals. All GMLR residuals have significant spatial autocorrelation with clustered residuals. On the contrary, all ESFR models are able to filter out spatial autocorrelation in the residuals, rendering insignificant Moran’s I. As we can see from the performance metrics, filtering spatial autocorrelation from the residuals substantially improves model fit and reduces model errors.

#### 3.3.3. Model Cross Validation

Cross validations were conducted to assess model overfitting and prediction accuracy. Table 9 shows that the MSE of the annual ESFR model is 19.2, 22.8% lower than that from GMLR (MSE = 24.9). For the seasonal models, the MSE of winter ESFR model is 12.5% lower than that of GMLR model. The MSE of the spring ESFR model is 14.0% lower than the GMLR model. The MSE of the summer ESFR model is 28.9% lower than the GMLR model. The MSE of the autumn ESFR model is 29.9% lower than the GMLR model.

**Figure 5:**
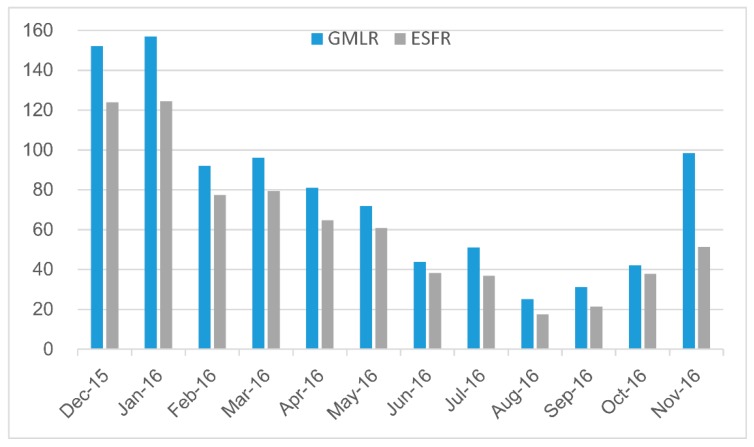
Monthly model leave-one-out cross validation results: test dataset MSE of GMLR and ESFR models.

For the monthly models (Figure 5), the MSE values of the GMLR models range from 25.2 to 157.0 and the mean is 78.5. The MSE values of the ESFR models range from 17.5 to 124.5 and the mean is 61.2. In all the monthly models, ESFR models have substantially lower cross validation errors than GMLR. It can be concluded that overall ESFR performs the best in the estimation of PM_2.5_ with the set of predictors where no monitoring stations exist.

### 3.4. Analysis of PM_2.5_ Concentrations Based on ESFR model

#### 3.4.1. PM_2.5_ Distribution Maps

**Figure 6:**
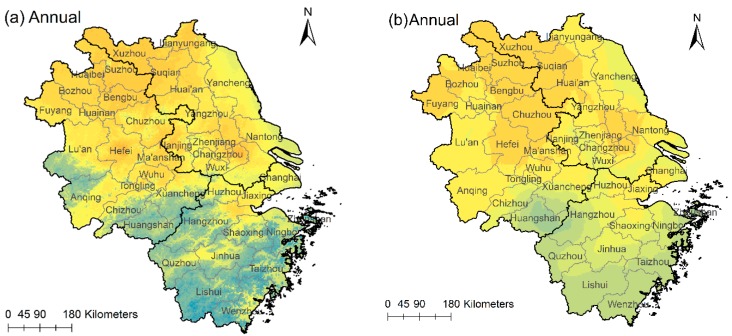

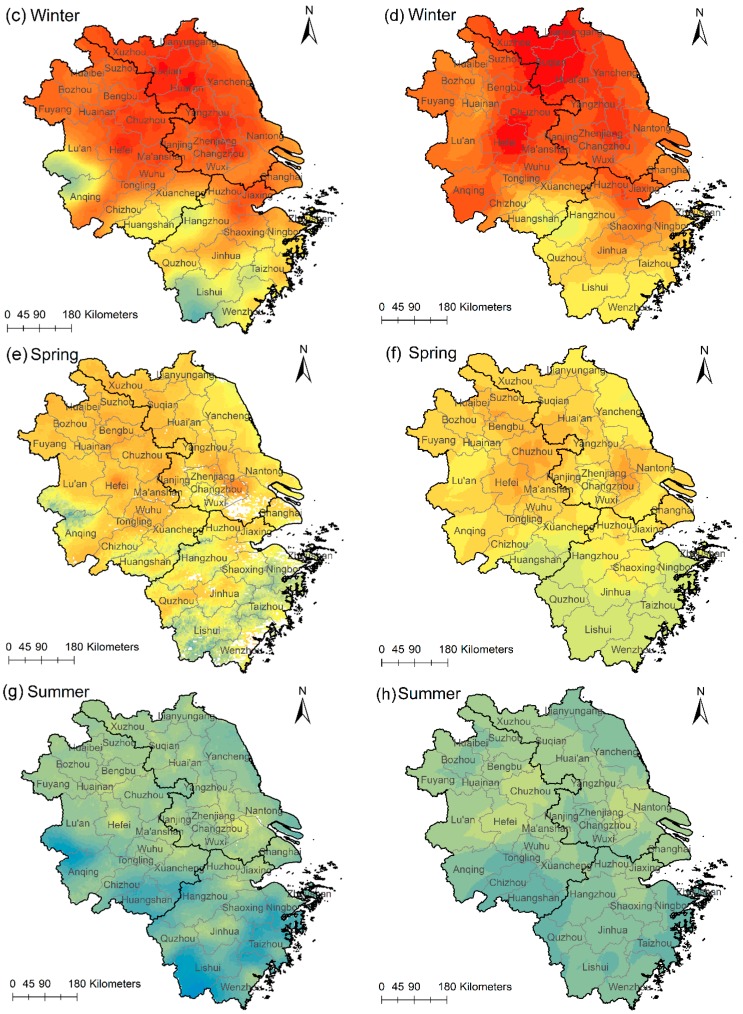

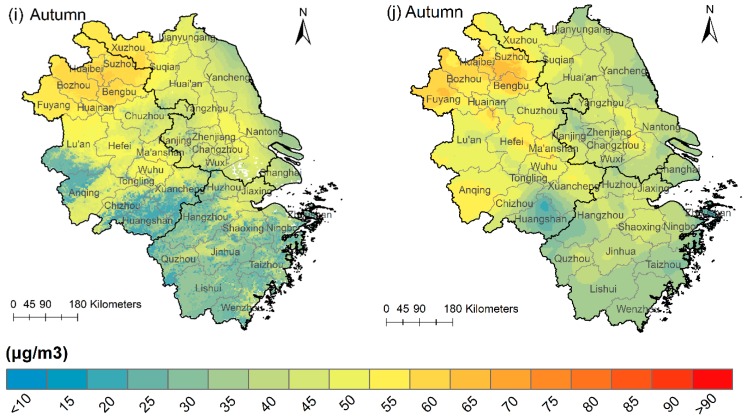
PM_2.5_ spatial distribution obtained from ESFR models (**a**,**c**,**e**,**g**,**i**) and Kriging interpolation of ground observations (**b**,**d**,**f**,**h**,**j**).

Figure 6a,c,e,g,i depict the annual and seasonal PM_2.5_ spatial distributions. They were derived from the ESFR models. Figure 6b,d,f,h,j were Kriging interpolations of observed PM_2.5_ at ground stations. It is obvious that PM_2.5_ spatial distributions based on ESFR models contain more details than the direct interpolations. For example, the junction of Lu’an and Anqing is an area with low PM_2.5_ concentration. However it is not reflected in the interpolation maps (Figure 6b,d,f,h,j).

Root Mean Square Error (RMSE) between model predictions and observations were calculated to evaluate the model accuracy. RMSE of the annual ESFR model is 4.1 μg/m^3^, 14.0% lower than the GMLR model (4.8 μg/m^3^). For the seasonal ESFR models, Summer model has the lowest RMSE (4.2 μg/m^3^), followed by autumn (RMSE = 4.9 μg/m^3^) and spring (RMSE = 6.3 μg/m^3^). The winter model has the highest RMSE (7.6 μg/m^3^). This error is acceptable for practical applications. All seasonal GMLR models have higher RMSE than the ESFR models. In ascending order, they can be sorted as summer (RMSE = 5.0 μg/m^3^), autumn (RMSE = 6.0 μg/m^3^), spring (RMSE = 7.0 μg/m^3^) and winter (RMSE = 8.4 μg/m^3^).

**Figure 7:**
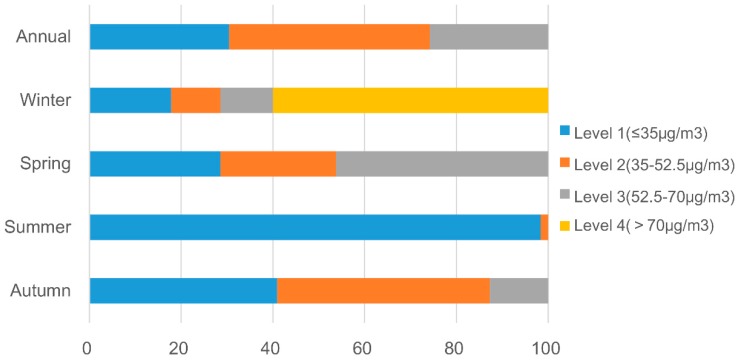
Area percentage of four levels in the whole year and four seasons.

Figure 6a provides an overview of PM_2.5_ pollution status in the YRD region. The annual mean of the estimated PM_2.5_ concentration is 40.0 μg/m^3^, lower than the observed mean value (51.3 μg/m^3^). We classified the PM_2.5_ concentrations into four levels: below the national standard (≤35 μg/ m^3^), 1.0–1.5 times of the standard (35–52.5 μg/m^3^), 1.5–2 times of the standard (52.5–70 μg/m^3^) and over twice of the standard (>70 μg/m^3^). We calculated the area percentages of each level and depicted them in Figure 7. For the whole year, 30.4% of the area has qualified air (level 1). In 43.7% of the area, the PM_2.5_ is at level 2. 25.8% of the area has level 3 PM_2.5_ pollution. Clearly, PM_2.5_ pollution in the YRD region is rather serious.

**Figure 8:**
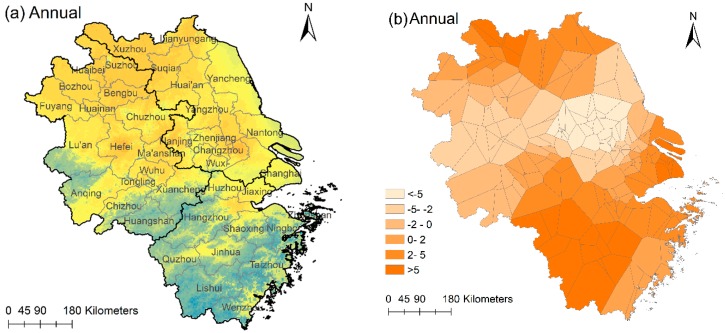

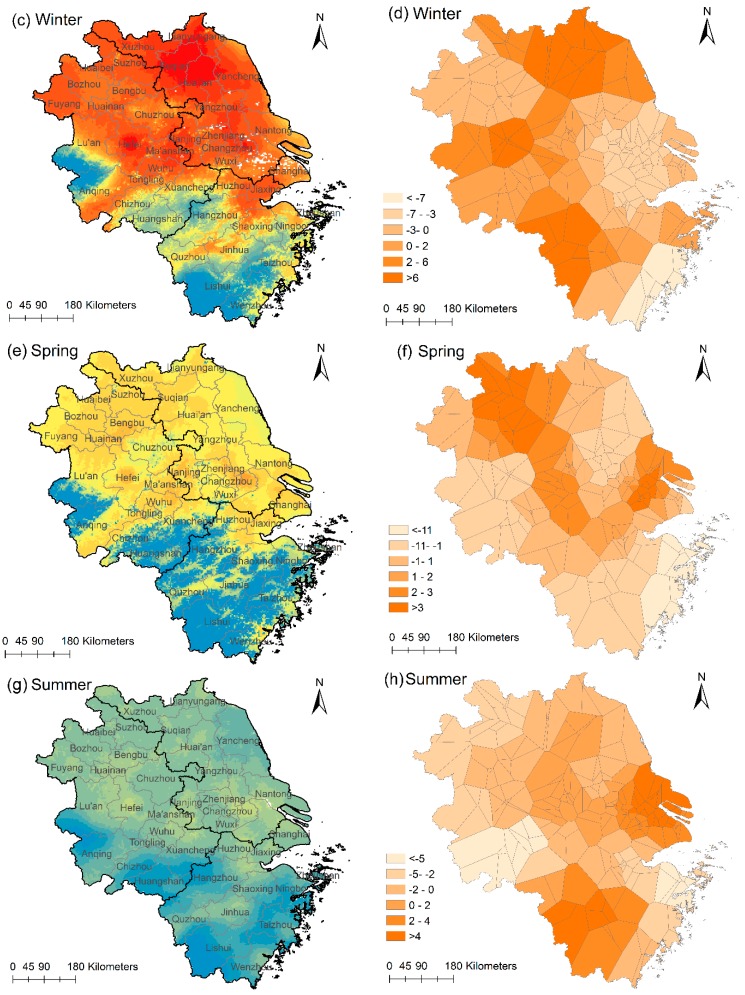

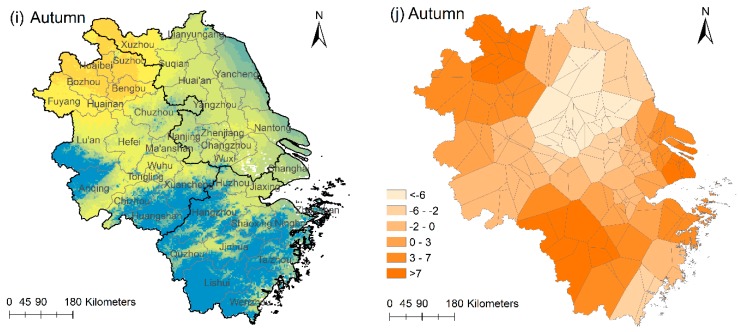
Annual and seasonal PM_2.5_ distribution maps (**a**,**c**,**e**,**g**,**i**) and eigenvector maps (**b**,**d**,**f**,**h**,**j**).

To demonstrate further the spatial effects on PM_2.5_ distribution, we calculated the linear combination of the eigenvectors, **E_k_β_k_**, for both the annual and seasonal models and visualized them in Figure 8b,d,f,h,j. The spatial patterns between each pair are strikingly similar because these eigenvector maps contain local spatial information of PM_2.5_ distribution. In Figure 8a, there is an obvious high-high cluster of PM_2.5_ concentration: Xuzhou-Suqian and it exactly corresponds to a highlighted area in the eigenvector map in Figure 8b. Besides, there is a low-low cluster region: Lishui-Taizhou in Zhejiang and it also corresponds to a highlighted area. For the seasonal maps, the situations are almost identical to these two cluster regions but there are differences. For example, in winter (Figure 8c), another high-high cluster appears in Hefei-Chuzhou and correspondingly in Figure 8d the **E_k_β_k_** is high in this area. In spring (Figure 8e), Changzhou-Nantong is high in PM_2.5_ concentration and it also has a relatively high value in Figure 8f. In autumn (Figure 8i), the high-high cluster expands from Xuzhou-Suqian to Xuzhou-Bengbu-Huaibei, and the highlighted area changes too (Figure 8j). The existence of high-high and low-low clusters of PM_2.5_ concentration shows that PM_2.5_ distribution is significantly influenced by spatial heterogeneity and spatial autocorrelation in YRD region and the spatial patterns change with time.

#### 3.4.2. PM_2.5_ Spatial-temporal Analysis in YRD region

The PM_2.5_ distribution maps show apparent spatial heterogeneity. The north of the YRD region has higher PM_2.5_ concentration than the south. Jiangsu Province has the highest annual mean PM_2.5_ concentration (50.3 μg/m^3^), followed by Shanghai (46.1 μg/m^3^), Anhui (42.6 μg/m^3^) and Zhejiang (23.7 μg/m^3^) in turn. Among all the prefecture-level cities, the top three with the highest PM_2.5_ concentration are Suqian (54.2 μg/m^3^), Huaibei (54.0 μg/m^3^) and Ma’an Shan (53.8 μg/m^3^). The bottom three are Lishui (10.0 μg/m^3^), Wenzhou (13.5 μg/m^3^) and Huangshan (18.5 μg/m^3^).

For different time scales, there exists similar spatial patterns of PM_2.5_ concentrations. Table 10 shows the top three cities with the best or the worst air quality in four seasons. The high concentration value always appears in the vicinity of Taizhou in Jiangsu, the junction area of Suqian and Huai’an, Hefei and Ma’anshan. The low concentration always clusters in the junction area of Lu’an and Anqing, Xuancheng, Hangzhou and several cities in southern Zhejiang province such as Lishui and Taizhou.

In the perspective of changes over time, PM_2.5_ average concentrations in the four seasons can be sorted in descending order as winter (65.2 μg/m^3^), spring (42.0 μg/m^3^), autumn (33.6 μg/m^3^) and summer (24.7 μg/m^3^). This tendency is consistent with the station monitoring data but the concentrations are undervalued for all the seasons. Besides, according to Figure 7, PM_2.5_ pollution is the severest in winter with 60.0% of the area having a level-4 PM_2.5_ pollution, which can be detrimental to human health. In spring, the percentage of area with unqualified PM_2.5_ concentration reaches up to 71.4% but the pollution levels are not as high as winter. The air is the best in summer, with 98.3% of the area above the national limit. The remaining 1.7% is at level 2 and has minor health consequence. Then in autumn the percentage of area with qualified PM_2.5_ concentration decreases to 40.9%.

#### 3.4.3. Pollution Sources Analysis

**Figure 9:**
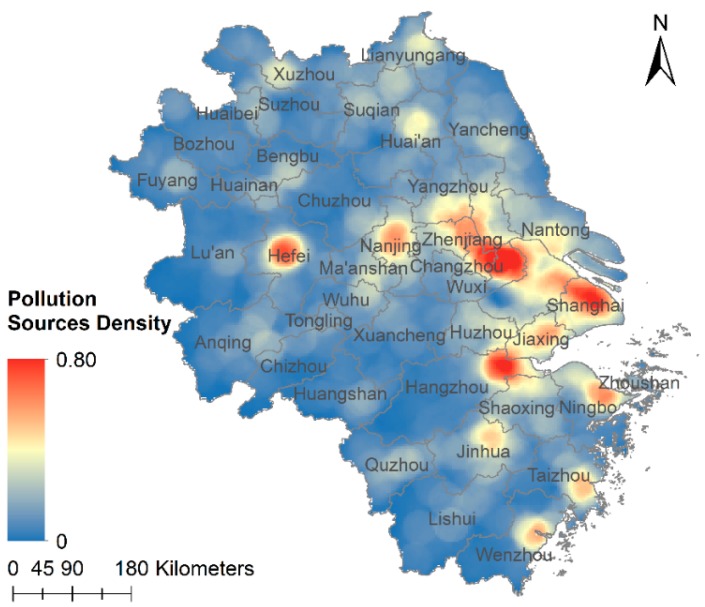
Pollution sources density map.

Figure 9 shows the density of likely pollution sources in the YRD region. The density value presents local causes for PM_2.5_ pollution to some degree. Shanghai, Changzhou, central Hefei, Jiaxing and the east of Hangzhou are regions with concentrated pollution sources, which can explain the relatively high PM_2.5_ concentrations in these areas. However, there are high PM_2.5_ concentration areas, such as Chuzhou and Anqing, with low pollution sources density. There are also low PM_2.5_ concentration areas with high pollution sources density, such as Taizhou in Zhejiang. These may be the results of particle dispersion influenced by temperature, pressure and so on. Overall, this is a simple quantitative way to find the main pollutant sources in a region, which will be helpful for air pollution treatment. More accurate predictions require modeling atmospheric transmissions, which is beyond the scope of this project.

## 4. Discussion

### 4.1. Spatial-Temporal Analysis of PM_2.5_ Concentrations Based on ESFR Models

In modeling ground PM_2.5_ concentrations, spatial autocorrelation is a main factor that limits the performances of conventional OLS models. In this paper, we developed eigenvector spatial filtering regression models, filtering spatial information from the regression residuals and representing it as a linear combination of selective eigenvectors of the spatial weights matrix in the model. In this way, residuals spatial autocorrelation is substantially reduced and model precision improved, enabling us to perform more accurate and reliable analyses and predictions.

The resulting models shed new lights on the relationships between PM_2.5_ and the atmospheric conditions, terrain, ground vegetation, and cultural features. By comparisons of models from different periods, we revealed the influences of the independent variables on PM_2.5_ and the variations of individual impacts over time, most of which are consistent with existing research findings. PBLH is negatively correlated with PM_2.5_ because Planetary Boundary Layer (PBL) can weaken the exchange between earth surface and free troposphere. The lower the PBLH is, the more particles are restricted near ground [56]. ST and RH pose different influence on PM_2.5_ in different periods as Table 5 and Table 6 show. As for ST, high temperature contributes to photochemical activity to produce more fine particles; however, it can also promote the convection of air and decrease PM_2.5_ concentrations. The relationship between RH and PM_2.5_ is also complex. When RH is low, PM_2.5_ concentration increases because of hygroscopic growth [57]. However, when RH is high enough, fine particles cluster together and fall to the ground, causing a decrease of PM_2.5_ concentrations in the air [16]. DEM is negatively correlated with PM_2.5_ because higher altitudes have good PM_2.5_ dispersion conditions [22]. PS has a positive correlation with PM_2.5_ concentration because high pressure can cause downdrafts and an accumulation of particles near the ground [58]. NDVI is not as important as reported in the literature, according to our study (Table 5 and Table 6). The PCCs in Table 2 and Table 3 also indicate a weak correlation between NDVI and PM_2.5_. In several models, NDVI shows a negative effect because vegetation can absorb PM_2.5_ emissions. In contrast, Fact_Den_ and Road_Den_ are positively correlated with PM_2.5_ concentrations, because they relate to the emission of pollutants. Road_Den_ is also removed from most of the models as transported emissions tend to move up to the boundary layer, hence are likely irrelevant to ground PM_2.5_ concentrations in our study area [25].

AOD is an important variable for modeling PM_2.5_ concentrations. On the whole as the annual model results in Table 5 show, AOD is positively correlated with PM_2.5_ concentration, because AOD tells how much sunlight is absorbed or scattered by aerosol particles. However, as we can see in most of the models, AOD has been removed for being not significant in the first stepwise procedure. We speculate a number of possible causes for this inconsistency. First, most studies on the relationship between AOD and PM_2.5_ are based on daily aggregates of stations located close to each other as a means to alleviate the missing data issue. As a result, AOD and PM_2.5_ may have significant relations on a daily time scale. This approach however may not work well for a longer duration because there are more chances of missing data due to atmospheric conditions. Second, the amount of missing data differs between stations, so the resulting means are biased. The relationship between PM_2.5_ and AOD weakened after calculating annual, seasonal and monthly PM_2.5_ averages [59]. If rudimentary interpolation and imputation are to be performed to estimate the missing data, excessive amount of errors will likely to be introduced. Besides, the large span of study area can also weaken their relationship due to the spatial heterogeneity. A previous study showed that there was a linear PM_2.5_-AOD relationship at one site in Italy but the results were different at other sites in Los Angeles and Beijing [60]. Applying more advanced methods of imputation to generate a more reliable AOD aggregates will lead to substantial improvement with the models.

Both in-sample fit and cross-validation show that ESFR model performs better than GMLR. Thus the ESFR model combined with remotely sensed data can be an effective way of estimating PM_2.5_ concentrations. PM_2.5_ distribution maps show that the northern YRD region has a higher concentration than the south. Jiangsu Province is the most seriously polluted among the four administrative regions, while Zhejiang is the cleanest. PM_2.5_ concentrations are the highest in winter and the lowest in summer. These spatial and temporal analyses can be verified by current researches and the station observations, indicating our ESFR model is a good approximation of PM_2.5_ processes in the region [38,61].

According to the ESF theory [47], spatial influence on PM_2.5_ distribution can be visualized by the linear combination **E_k_β_k_**. In our case study of the YRD region, although we had different eigenvectors for the annual and seasonal models and linear combination of eigenvectors also varied with time, there are similar spatial patterns revealed by the eigenvector maps. By comparing with the PM_2.5_ distribution maps, we found a corresponding relationship between high values of **E_k_β_k_** and the clusters of PM_2.5_ concentrations. This geographic pattern prevails in the YRD region. In addition, high-high or low-low PM_2.5_ clusters such as Xuzhou-Suqian and Lishui-Wenzhou were found. This is another supporting evidence that ESFR model can effectively uncover spatial structures using eigenvector maps, which is a unique advantage of the ESF approach.

### 4.2. Real-Time Monitoring of Ground PM_2.5_ Concentrations

In addition to long term PM_2.5_ analysis as shown in the annual, seasonal and monthly modeling of the YRD region, the ESFR model provides a way of real-time monitoring of ground PM_2.5_ concentrations. In the previous ESFR PM_2.5_ models [51], the independent variables are observations of other air pollutants, including PM_10_, SO_2_, NO_2_, CO and O_3_, which are collected from the same monitoring stations as PM_2.5_. They have the same resolutions as PM_2.5_ and are of limited relevance in the estimation of PM_2.5_ on a finer spatial or temporal scale. Besides, current methods of obtaining PM_2.5_ concentration data are through ground measurements at fixed stations or portable PM_2.5_ detectors, which can be time consuming and costly, particularly if greater spatial and temporal resolutions are required. In our ESFR model, most of the covariates are remotely sensed data with increasing spatial and temporal resolutions, such as AOD, ST, PS, PBLH and so on. Considering that ESFR model has a good prediction ability, we can construct ESFR models based on real time remotely sensed data and provide short-term or near real-time estimations of PM_2.5_ concentrations for a given region, enabling more effective air quality management and more timely environmental guidance for daily activities.

### 4.3. Limitations and Future Enhancements

Missing observations remain a major problem with AOD, which may well be a reason for the weak association with PM_2.5_ in the model. In addition to cloud cover, the dark target algorithm has trouble in retrieving AOD in some urban areas. The daily and monthly models are affected the most due to a low AOD coverage. Our future work is to combine AOD products from various sources into a database with large spatial coverage [28] and high precision so that it can be widely used in this field. In addition, we will further improve the model precision experimenting with different specifications of spatial weights matrix and by exploring additional covariates. As the distribution of PM_2.5_ is influenced not only by spatial autocorrelation but also time effects, an integrated spatio-temporal regression model should be the ultimate approach to modeling air pollution [62], where the eigenvector spatial filter is a promising direction [50,63]. Lastly, atmospheric transmission and dynamics have yet to be incorporated in the current modeling process, which is clearly a limitation, though such effects were alleviated to some extent because the study area is large and the temporal span is year long hence the variables can be seen as stable and representative in the study area.

## 5. Conclusions

As PM_2.5_ pollution becomes increasingly severe, it is necessary to develop accurate and reliable models for studying the spatial and temporal characteristics of ground PM_2.5_. The contribution of this paper is two-fold. On the one hand, we develop an ESFR model for ground PM_2.5_ concentrations estimation. Spatial influence is incorporated to classic OLS models and performances are substantially improved. Spatial characteristics of PM_2.5_ concentrations are effectively shown in the model specification. On the other hand, models with improved accuracies combined with remotely sensed data offer an effective means to estimate PM_2.5_ concentrations over time and space. High resolution and real-time PM_2.5_ distribution maps can be generated and provide detailed analysis results for chronic and epidemiological studies. However, some of the covariates used in our model are not significant as reported by previous studies. Data with higher quality will be explored and applied in our next study to further analyze spatial-temporal characteristics of ground PM_2.5_ concentrations. The spatial differences in the relationships between influential factors and PM_2.5_ are not shown in the model. As the next step, we will conduct GWR and extract location varied coefficients from ESF so as to analyze the coefficients’ spatial variations.

## Figures and Tables

**Table 1:** Summary of dataset.

Items	PM_2.5_ (μg/m^3^)	AOD (10^−3^)	ST (K)	PS (hPa)	RH (%)	PBLH (m)	NDVI (%)	Elevation (m)
Min	23.2	−5.0	270.2	912.9	45.8	132.2	−19.6	−92
Max	67.9	4952.0	327.8	1029.6	93.4	1013.5	99.9	1922
Mean	51.3	540.8	292.0	1001.3	69.7	389.7	62.0	138.3
Std.dev	7.9	278.3	13.5	22.1	0.8	140.6	1.9	232.0

**Table 2:** Annual and Seasonal Pearson Correlation Coefficients between PM_2.5_ and other covariates.

Time	AOD	PBLH	PS	RH	ST	NDVI	DEM	Fact_Den_	Road_Den_
Annual	0.303 **	−0.395 **	0.560 **	−0.407 **	−0.452 **	−0.155 *	−0.320 **	0.257 **	0.138 *
Winter	0.139 *	−0.373 **	0.620 **	−0.383 **	−0.487 **	−0.079	−0.385 **	0.272 **	0.185 **
Spring	0.403 **	−0.187 **	0.496 **	−0.318 **	0.163 *	−0.132	−0.369 **	0.326 **	0.201 **
Summer	0.248 **	−0.103	0.366 **	0.007	0.088	−0.137 *	−0.216 **	0.384 **	0.289 **
Autumn	−0.103	−0.559 **	0.300 **	−0.378 **	−0.569 **	−0.130	−0.049	0.001	−0.151 *

* Significant at α = 0.05 (Two-tailed); ** Significant at α = 0.01(Two-tailed).

**Table 3:** Monthly Pearson Correlation Coefficients between PM_2.5_ and other covariates.

Time	AOD	PBLH	PS	RH	ST	NDVI	DEM	Fact_Den_	Road_Den_
15 Dec	0.099	−0.248 **	0.614 **	−0.038	−0.322 **	−0.148 *	−0.415 **	0.340 **	0.303 **
16 Jan	0.100	−0.058	0.662 **	−0.550 **	−0.364 **	0.164	−0.517 **	0.275 **	0.212 *
16 Feb	0.312 **	−0.472 **	0.437 **	−0.190 **	−0.509 **	−0.155 *	−0.207 **	0.267 **	0.076
16 Mar	0.321 **	−0.511 **	0.227 **	−0.230 **	−0.234 **	−0.117	−0.085	0.179 **	0.049
16 Apr	0.428 **	−0.265 **	0.542 **	−0.497 **	0.198 **	0.065	−0.398 **	0.320 **	0.246 **
16 May	0.230 **	0.095	0.461 **	−0.079	0.146 *	−0.061	−0.398 **	0.234 **	0.243 **
16 Jun	0.117	0.037	0.389 **	0.006	−0.044	−0.109	−0.313 **	0.390 **	0.316 **
16 Jul	0.330 **	−0.217 **	0.421 **	0.212 **	0.235 **	−0.231 **	−0.329 **	0.417 **	0.374 **
16 Aug	0.224 **	−0.509 **	0.070	0.228 **	−0.395 **	−0.094	0.150 *	0.116	−0.053
16 Sep	0.264 **	−0.466 **	0.333 **	−0.544 **	0.070	−0.113	−0.059	0.222 **	0.034
16 Oct	−0.244 **	−0.549 **	0.230 *	−0.026	−0.498 **	−0.095	−0.111	0.039	−0.113
16 Nov	−0.074	−0.457 **	0.405 **	−0.267 **	−0.591 **	−0.200 **	−0.180 *	−0.023	−0.182 *

* Significant at α = 0.05 (Two-tailed); ** Significant at α = 0.01 (Two-tailed).

**Table 4:** Annual, Seasonal and monthly mean PM_2.5_ Moran’s I.

Time	Moran’s I	*p*-Value	Time	Moran’s I	*p*-Value
Annual	0.563	<0.001	16 Apr	0.526	<0.001
Winter	0.549	<0.001	16 May	0.296	<0.001
Spring	0.494	<0.001	16 Jun	0.361	<0.001
Summer	0.416	<0.001	16 Jul	0.399	<0.001
Autumn	0.610	<0.001	16 Aug	0.539	<0.001
15 Dec	0.547	<0.001	16 Sep	0.526	<0.001
16 Jan	0.524	<0.001	16 Oct	0.564	<0.001
16 Feb	0.495	<0.001	16 Nov	0.571	<0.001
16 Mar	0.598	<0.001			

**Table 5:** Standardized coefficients and *p*-values of annual and seasonal PM_2.5_ ESFR Model.

Variables	Annual		Winter		Spring		Summer		Autumn	
	Beta	*p*	Beta	*p*	Beta	*p*	Beta	*p*	Beta	*p*
AOD	0.17	0.01	0.06	0.24	/	/	/	/	0.08	0.10
ST	−0.46	0.00	−0.23	0.02	0.18	0.00	/	/	/	/
PS	0.68	0.00	0.56	0.00	/	/	0.40	0.00	0.43	0.00
RH	0.31	0.00	0.32	0.00	/	/	−0.53	0.00	0.16	0.10
PBLH	−0.66	0.00	−0.32	0.00	−0.32	0.00	−0.59	0.00	−0.94	0.00
NDVI	−0.10	0.01	/	/	/	/	−0.14	0.01	/	/
DEM	−0.24	0.00	−0.16	0.05	−0.38	0.00	/	/	−0.30	0.00
Fact_Den_	0.31	0.00	0.20	0.00	0.43	0.00	0.41	0.00	/	/
Road_Den_	/	/	/	/	/	/	/	/	/	/
R^2^_adj_	0.70		0.64		0.49		0.51		0.65	
AICc	1255.8		1503.4		1357.0		1260.5		1314.5	
MSE	19.2		66.4		43.9		18.9		27.4	

AICc denotes Corrected Akaike Information Criterion; MSE denotes the Mean Square Error of leave-one-out cross validation (LOOCV).

**Table 6:** Standardized coefficients and *p*-values of monthly PM_2.5_ ESFR Model.

**Variables**	**15 Dec**		**16 Jan**		**16 Feb**		**16 Mar**		**16 Apr**		**16 May**	
	**Beta**	***p***	**Beta**	***p***	**Beta**	***p***	**Beta**	***p***	**Beta**	***p***	**Beta**	***p***
AOD	/	/	/	/	/	/	/	/	/	/	/	/
ST	−0.72	0.00	/	/	0.15	0.33	0.34	0.00	/	/	0.13	0.10
PS	0.59	0.00	0.63	0.00	0.32	0.00	/	/	/	/	0.22	0.08
RH	0.31	0.00	/	/	0.80	0.00	0.17	0.09	−0.36	0.01	−0.21	0.09
PBLH	0.36	0.00	−0.39	0.00	−0.97	0.00	−0.66	0.00	−0.10	0.57	/	/
NDVI	−0.07	0.20	/	/	/	/	/	/	−0.09	0.08	/	/
DEM			−0.19	0.06			−0.28	0.00	−0.30	0.00	−0.26	0.01
Fact_Den_	0.26	0.00			0.14	0.02	0.34	0.00	0.39	0.00	0.32	0.00
Road_Den_	/	/	0.24	0.01	/	/	/	/	/	/	/	/
R^2^_adj_	0.60		0.63		0.57		0.55		0.54		0.37	
AICc	1367.1		791.3		1477.0		1534.6		1485.1		1456.6	
MSE	124.0		124.5		77.4		79.4		64.8		60.9	
**Variables**	**16 Jun**		**16 Jul**		**16 Aug**		**16 Sep**		**16 Oct**		**16 Nov**	
	**Beta**	***p***	**Beta**	***p***	**Beta**	***p***	**Beta**	***p***	**Beta**	***p***	**Beta**	***p***
AOD	−0.11	0.10	0.12	0.07	/	/	0.16	0.00	−0.12	0.10		
ST	/	/	/	/	/	/	/	/	/	/	−0.84	0.00
PS	0.50	0.00	0.24	0.00	0.25	0.02	/	/	/	/	0.50	0.00
RH	/	/	−0.52	0.00	/	/	/	/	/	//	0.30	0.00
PBLH	0.35	0.00	−0.24	0.01	−0.58	0.00	−0.53	0.00	−0.40	0.00	/	/
NDVI	−0.10	0.10	−0.14	0.01	/	/	−0.12	0.01	/	/	/	/
DEM	/	/	/	/	/	/	/	/	−0.41	0.00	−0.28	0.00
Fact_Den_	0.41	0.00	0.37	0.00	0.14	0.01	0.18	0.00	0.13	0.06	0.16	0.00
Road_Den_	/	/	/	/	/	/	0.12	0.10	/	/	/	/
R^2^_adj_	0.36		0.49		0.55		0.61		0.51		0.73	
AICc	1218.3		1391.5		1170.8		1238.4		767.0		1264.5	
MSE	38.2		36.8		17.5		21.5		37.9		51.3	

**Table 7:** Annual and seasonal ESFR model evaluation indicators.

Time	Adj. R^2^		RSE		MAPE		AICc	
	GMLR	ESFR	GMLR	ESFR	GMLR	ESFR	GMLR	ESFR
Annual	0.60	0.70	4.85	4.24	7.70	6.66	1307.1	1255.8
Winter	0.57	0.64	8.58	7.86	8.84	7.85	1530.6	1503.4
Spring	0.39	0.49	7.06	6.46	9.91	8.94	1384.0	1357.0
Summer	0.31	0.51	5.10	4.27	13.61	10.64	1331.3	1260.5
Autumn	0.48	0.65	6.12	5.06	11.77	9.19	1384.8	1314.5

**Table 8:** Residual Moran’s I of annual, seasonal and monthly models.

Time	GMLR		ESFR	
	Moran’s I	*p*-Value	Moran’s I	*p*-Value
Annual	0.101	<0.001	−0.057	0.970
Winter	0.097	<0.001	−0.060	0.976
Spring	0.111	<0.001	−0.021	0.709
Summer	0.278	<0.001	−0.004	0.494
Autumn	0.224	<0.001	−0.022	0.730
15 Dec	0.165	<0.001	−0.036	0.841
16 Jan	0.116	0.001	−0.038	0.769
16 Feb	0.077	0.002	−0.061	0.976
16 Mar	0.104	<0.001	−0.037	0.877
16 Apr	0.206	<0.001	−0.008	0.544
16 May	0.162	<0.001	0.011	0.282
16 Jun	0.144	<0.001	−0.020	0.693
16 Jul	0.254	<0.001	0.006	0.340
16 Aug	0.312	<0.001	−0.060	0.974
16 Sep	0.314	<0.001	−0.034	0.848
16 Oct	0.095	0.002	−0.053	0.887
16 Nov	0.376	<0.001	−0.067	0.980

**Table 9:** Annual and seasonal model cross validation results.

Time	GMLR	ESFR
Annual	24.9	19.2
Winter	75.8	66.4
Spring	51.0	43.9
Summer	26.6	18.9
Autumn	39.1	27.4

**Table 10:** The top three cities with the best or the worst air quality.

Season	Worst			Best		
	1st	2nd	3rd	1st	2nd	3rd
Winter	Suqian (90.8)	Huai’an (88.5)	Lianyungang (86.6)	Lishui (11.2)	Wenzhou (23.3)	Huangshan (35.0)
Spring	Huainan (59.2)	Bengbu (59.1)	Huaibei (61.4)	Lishui (9.3)	Wenzhou (14.8)	Huangshan (15.9)
Summer	Wuxi (32.4)	Huainan (31.6)	Bengbu (30.9)	Lishui (11.1)	Huangshan (14.1)	Wenzhou (17.4)
Autumn	Huaibei (55.6)	Bozhou (55.3)	Bengbu (54.6)	Lishui (7.5)	Wenzhou (8.2)	Huangshan (11.0)

Note: The number in brackets is the mean PM_2.5_ concentration (units: μg/m^3^).
